# *Axonopus graniticola*, a new species of *A*. ser. *Suffulti* (Poaceae, Panicoideae, Paspaleae) from Minas Gerais, Brazil

**DOI:** 10.3897/phytokeys.21.4157

**Published:** 2013-03-22

**Authors:** Pedro Lage Viana, Luiza Fonseca Amorim de Paula

**Affiliations:** 1 Bicho do Mato Instituto de Pesquisa, Rua Perdigão Malheiros 222, 30380-234, Belo Horizonte, Minas Gerais, Brazil; 2 Departamento de Botanica, Instituto de Ciencias Biológicas, Universidade Federal de Minas Gerais, 31270-901, Belo Horizonte, Minas Gerais, Brazil

**Keywords:** Atlantic Forest, *inselbergs*, Grasses, new taxon, Gramineae

## Abstract

A new species of *Axonopus* ser. *Suffulti* from Minas Gerais, Brazil, is described and illustrated. Comparison with morphologically related species, as well as comments on the ecology and the conservation status are provided.

## Abstract (pt)

Uma nova espécie de *Axonopus* ser. *Suffulti* é descrita e ilustrada para o estado de Minas Gerais, Brasil. São fornecidas comparações com espécies morfologicamente relacionadas, assim como comentários sobre ecologia e estado de conservação.

## Introduction

*Axonopus* P.Beauv. is an American genus of Poaceae comprising approximately 110 species (Black 1963, Clayton and Renvoize 1986, Cialdella et al. 2006). Most of its species occur in the tropics, where it is especially diverse in the Neotropical savannas, such as the Brazilian *cerrado* (Mendonça et al. 1998), the Espinhaço Range mountains (Viana and Filgueiras 2008), the Amazonian savannas and the Guayana Shield (Davidse et al. 2004). Traditionally, *Axonopus* was included in a wide circumscription of the tribe Paniceae, Panicoideae subfamily (e.g. Black 1963, GPWG 2001, Cialdella et al. 2006, Giraldo-Cañas 2007, 2008). However, the identity of Paniceae s.l. was challenged in the latest proposal of classification of this tribe (Morrone et al. 2012), based on an integrated analysis of *ndhF* plastid DNA and morphology. The authors split Paniceae s.l. into Paniceae s.s. (pantropical, basic chromosome number × = 10) and Paspaleae (American, × = 9), the latter encompassing *Axonopus* and other 38 genera.

Chase (1911) recognized three sections of *Axonopus*: *Axonopus* sect. *Cabrera* (Lag.) Chase, *Axonopus* sect. *Lappagopsis* (Steud.) Chase, and *Axonopus* sect. *Axonopus*, a circumscription that was followed by Dedecca (1956) in his revision of the genus for Brazil. Thereafter, Black (1963), in his taxonomic study of the genus, divided *Axonopus* sect. *Axonopus* into the series *Axonopus*, *Barbigeri*, *Cappilares*, *Fastigiati*, and *Suffulti*, based on such combination of characters as life span, indumentum, the number of nerves in the upper glume, trichomes in the rachis of racemes, and the color of fertile florets. The only attempt to assess the monophyly of Black’s *Axonopus* infrageneric groups using a combined analysis of morphological and molecular data (López and Morrone 2012) do not support Black’s classification. Some groups, however, appear to be monophyletic, like serie *Suffulti*. Nonetheless, a comprehensive phylogeny including a broader sampling within *Axonopus* is necessary to support a robust infrageneric classification of the genus.

Species of the *Axonopus* ser. *Suffulti* are perennial plants, with the upper glume and lower lemma lacking a central nerve, and fertile florets characteristically shiny brown to dark brown (Black 1963, Cialdella et al. 2006). Cialdella et al. (2006) published a comprehensive revision of the taxon, providing detailed descriptions of the 16 accepted names, ornamentation of the upper floret on SEM, illustrations, a key for identification of the species, and nomenclatural updates. Fifty five species of *Axonopus* are currently indicated to Brazil (Filgueiras and Rodrigues 2012), and seven of them are placed in the *Axonopus* ser. *Suffulti*.

A floristic survey in an overlooked granitic outcrop, or *inselberg*, in northeastern Minas Gerais, Brazil (de Paula et al. in prep.), revealed at least five new species of flowering plants. One of those, belonging to the *Axonopus* ser. *Suffulti*, is herein presented, illustrated and compared with putatively related species. SEM images of the fertile floret, as well as comments on its ecology and the conservation status are provided. For SEM images, samples were mounted on stubs, coated with gold palladium in a Hummer 6.2 (Anatech, Union City, CA, USA) sputtering system and viewed with a JSM-541OLV (JEOL, Tokyo, Japan) at 10kV.

## Taxonomic treatment

### Axonopus graniticola

P.L.Viana sp. nov.

urn:lsid:ipni.org:names:77126079-1

http://species-id.net/wiki/Axonopus_graniticola

#### Diagnosis.

*Axonopus graniticola* is distinguished from all other species of the *Axonopus* ser. *Suffulti* by its mostly caulinar leaves, distichous laterally compressed leaf sheaths, 1.5–2.5 cm wide leaf blades, deciduous with a subcordate base, and multi-racemose inflorescences of 26–75 racemes, with the basal ones re-branched.

#### Type.

**Figure 1.**
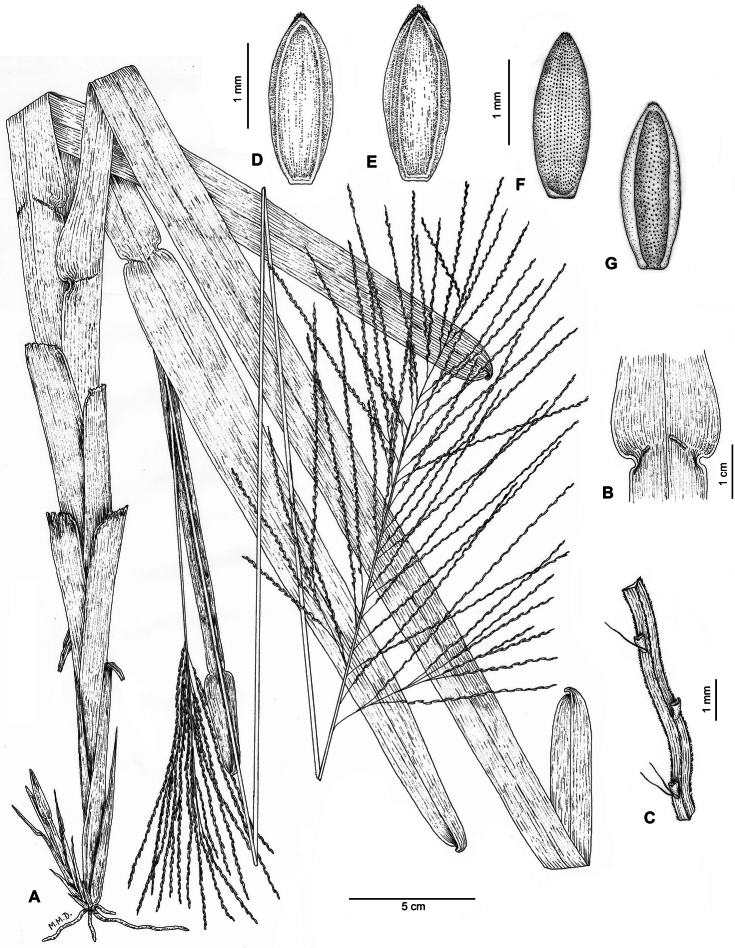
*Axonopus graniticola* P.L. Viana **A** Habit **B** Ligular region, adaxial side **C** Rachis of receme **D **Spikelet, upper glume view **E** Spikelet, lower lemma view **F** Upper floret, lemma view **G** Upper floret, palea view. Drawn from the holotype (de Paula et al. 145).

**Figure 2.**
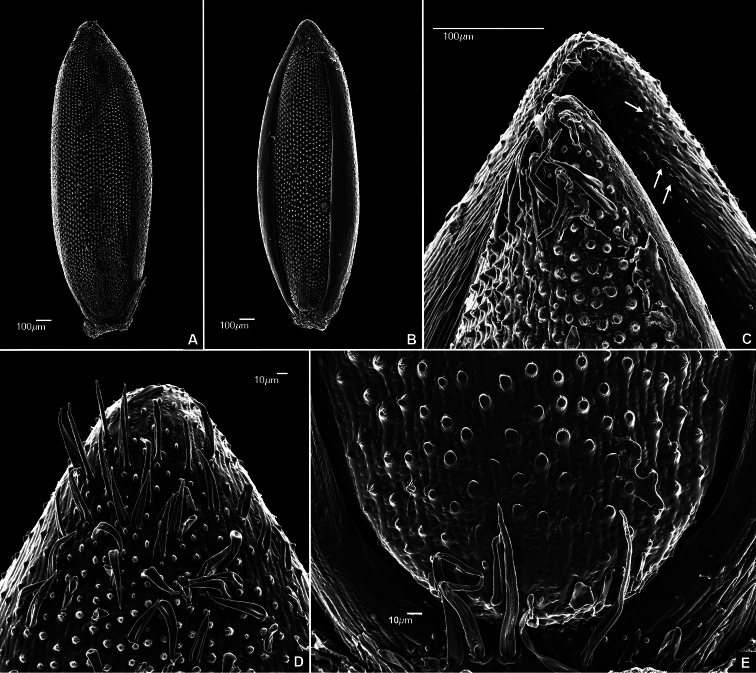
SEM micrographs of upper floret **A** Lemma view **B** Palea view **C** Apical portion of floret showing silica bodies (arrows) **D** Upper lemma apex, showing conspicuous macrohairs and pappilae **E **Basal portion of floret. Images taken from the holotype (de Paula et al. 145).

**Figure 3.**
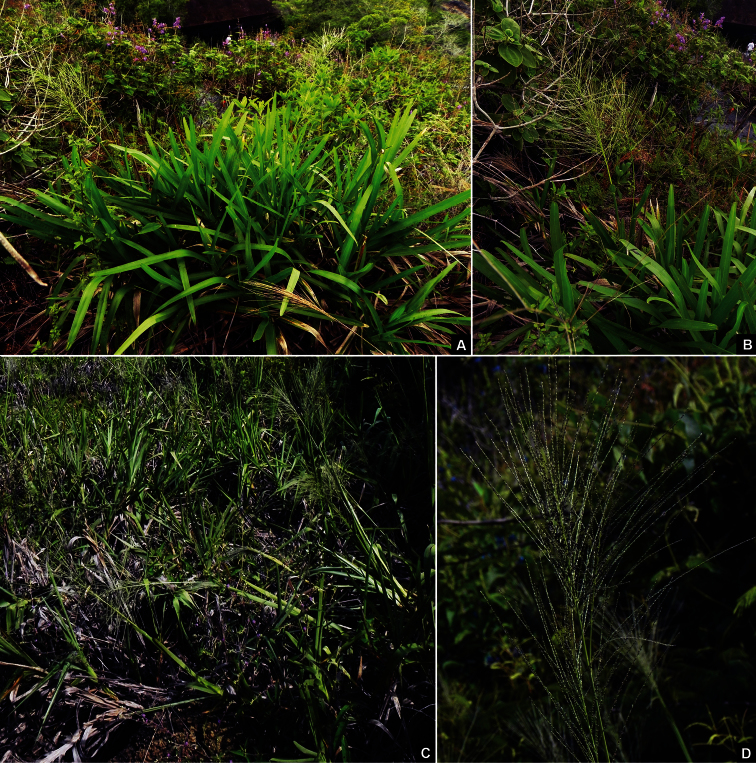
In situ photographs of *Axonopus graniticola* P.L. Viana **A** Dense clump of A. graniticola during the rainy season **B** Flowering culm **C** Clumps, in the beginning of the dry season **D** Detail of panicle. Photographs by L.F.A. de Paula.

**BRAZIL:** Minas Gerais: Município Teófilo Otoni, Afloramento rochoso ao lado esquerdo da MG 418, cerca de 30 km norte de Teófilo Otoni, 17°51'21.5"S, 41°15'39.4"W, 560 m alt., 8 Jan 2011, L.F.A. de Paula, N.F.O. Mota, P.L. Viana, T.B. Jorge, P.M. Burkowski 145 (Holotype: BHCB! Isotypes: RB! NY!) (Figures 1–3).

#### Description.

Plants perennial, densely caespitose, with very short falciform rhizomes. Culms 95–125 cm long, erect to decumbent, slightly curved at the base, not geniculate, unbranched; nodes various, hidden by leaf sheaths, glabrous; internodes 5–8.5 mm wide, cylindrical to slightly flattened, glabrous, stramineous. Leaves distichous, mostly caulinar; leaf sheaths 5.5–32 cm long, larger than the internodes, conduplicate, strongly keeled, striate, scabrous, glabrescent, persistent; ligule 0.15–0,20 mm long or absent, ciliate, apparently deciduous, because it is usually absent in older leaves; collar prominent, glabrous; leaf blades (4.5)12–32 × 1.5–2.5 cm, oblong to linear, lanceolate, flat, retrosely scabrous abaxially, antrorselly scabrous adaxially, eventually with sparse hairs on abaxial or adaxial surfaces, deciduous, nerves prominent, margins scabrous, base rounded, subcordate, arising from a constriction of 1–2 mm long in each margin of the ligular region, apex obtuse, asymmetrical, emarginate, slightly folding, reflexed, scabrous. Inflorescences 2 per flowering culm, terminal and axillary; peduncle up to 55 cm long, partially included in the leaf sheaths, cylindrical to angulose, striate, scabrous; pulvinulus pubescent; main axis 8–16.5 × 0.05–0.14 cm, angulose, striate, scabrous; panicles 12–26 cm long, in dense clusters of alternate to verticillate racemes, the lower branches re-branching in 5–18 racemes; racemes (4–)9–16.5 cm long, the apical ones slightly shorter than the basal, 26–75 per panicle; rachis of racemes triquetous, fertile all along, except for the 1–4.5 mm basal portion length, ending in a fertile spikelet, (5)10–15 spikelets per portion of 25 mm long, pubescent, scabrous in the angles; pedicels 0.25–0.5 mm long, scaberulous, sometimes with a few hyaline tuberculate trichomes to 0.8–1.5 mm long. Spikelets 1.8–2.0 × 0.6–0.8 mm, oblong-ellipsoid, dorsiventrally compressed, apex acute; upper glume as long as the spikelet, elliptical, membranous, glabrous or with sparse trichomes, hyaline to stramineous, 2–4(–5)-nerved, nerves prominent, scaberulous in the apex, mid-nerve occasionally present; lower lemma glumiform, 2(–3)-nerved, nerves glabrous; upper lemma 1.8–2.0 × 0.8–0.9 mm, elliptical, stiff, glabrous, except for a discrete tuft of short white hairs at the apex, densely ornamented by diminute papillae, fading in density toward the margins, shiny brown to dark brown, apex acute, brown to pale, base brown to pale; upper palea similar to the upper lemma but slightly shorter, 1.7–1.9 × 0.6–0.8 mm, glabrous. Lodicules 0.2–0.3 mm long, 2, oblong, erose; stamens 3, anthers ca. 0.8 mm, dorsifixed, purplish; stigmas plumose, whitish. Caryopsis not seen.

#### Etymology.

The epithet refers to the occurrence of the plants of this species on exposed granite rock outcrops.

#### Morphological comments.

The new species has perennial habit, scabrous pubescent rachis, with few scattered trichomes 0.8–1.5 mm long associated with the region of the pedicel insertion, the 2–4(–5)-nerved upper glume and 2(–3)-nerved lower lemma, and typically shiny brown and stiff fertile florets. In accordance with Black’s (1963) infrageneric circumscription of *Axonopus*, accepted by Cialdella et al. (2006) and Giraldo-Cañas (2007, 2008), and the combination of the above features, *Axonopus graniticola* should be placed into the *Axonopus* subg. *Axonopus* ser. *Suffulti*.

A mid-nerve in the upper glume and lower lemma is found in some spikelets of the specimen *de Paula 237*. Although this feature is uncommon in *Axonopus* ser. *Suffulti* (Cialdella et al. 2006), some species included in this group can bear a discrete mid-nerve in these bracts in some spikelets. For example, in the delicate Peruvian *Axonopus elegantulus* (J. Presl) Hitchc. and in *Axonopus flabeliformis* Swallen, from northern South America, some spikelets can have the upper glume with a visible mid-nerve; in the Venezuelan *Axonopus magallanesiae* Giraldo-Cañas, both upper glume and lower lemma can be 4-5-nerved, with a noticeable mid-nerve on the bracts (Cialdella et al. 2006), as recorded in some spikelets of the new species.

*Axonopus flabeliformis* shares with *Axonopus graniticola* the characteristically equitant base, with distichous, laterally compressed and persistent leaf sheaths, disposed along the culm. The compound panicle, occasionally occurring in *Axonopus flabeliformis*, and spikelets 1.6–2.2 mm long, also suggest affinity, even though superficial, to the new species. *Axonopus graniticola* can be easily distinguished from *Axonopus flabeliformis* by its wider leaf blades (1.5–2.5 cm vs. 0.5–0.9 cm in *Axonopus flabeliformis*), the rounded and subcordate base of the blade arising from a constriction in the ligular region (against blade bases straight and following the sheath apex width in *Axonopus flabeliformis*), and its multi-flowered panicles (26–75 racemes vs. 6–20(–30) racemes in *Axonopus flabeliformis*).

The new species also bears slight resemblance to *Axonopus pressus* (Nees ex Steud.) Parodi, from the Brazilian *cerrado*, Bolivia and Paraguay, by its strongly conduplicate and keeled leaf sheaths, giving the typical laterally compressed aspect to the plant. However, the leaves of *Axonopus pressus* are predominantly basal, contrasting with mostly caulinar leaves of *Axonopus graniticola*, with shorter spikelets (1.8–2 mm long, vs. 2.2–3 mm in *Axonopus pressus*), wider leaf blades (1.5–2.5 cm, vs. 0.8–1.2 cm in *Axonopus pressus*) and inflorescences with 26–75 racemes (against less than 35 racemes in *Axonopus pressus*). Moreover, the panicles of the new species are compound, with the lower branches re-branching in 5–18 racemes, a feature absent in *Axonopus pressus*, with its panicles with unbranched racemes.

The flat and characteristically wide leaf blades and the compound panicles of the new species bear a slight resemblance to the widely distributed *Axonopus scoparius* (Flüggé) Kuhlm. However, the latter species is placed in the *Axonopus* sect. *Axonopus* ser. *Barbigeri* Black (Black 1963, Giraldo-Cañas 2007, 2008), and is characterized, among others, by spikelets with the upper glume and lower lemma with a central nerve, and pale brown upper florets. *Axonopus graniticola* plainly fits the circumscription of the *Axonopus* sect. *Axonopus* ser. *Suffulti*, as discussed above.

#### Ornamentation of fertile floret on SEM.

(Figure 2). Abaxial surface of palea and lemma ornamented with papillae, silica bodies, macro-hairs and micro-hairs. Papillae simple, conical, apex acute, one per cell, evenly distributed in longitudinal rows on the floret surface, except in the margins of the lemma, which lack papillae. Silica bodies equidimensional, dumbbell shaped, visible on the apical portion of lemma margins. Macro-hairs unicellular, simple, located in the apex of lemma and palea and in the basal portion of the lemma (Figure 2E). Micro-hairs collapsed in the studied material, probably due to samples preparation process, distributed in the apex of lemma and palea and in the basal portion of the lemma.

The presence of numerous papillae, dumbbell shaped equidimensional silica bodies on the apex of fertile floret and macro- and micro-hairs on the base and apex of floret are typical features of species included in *Axonopus* ser. *Suffulti* (Cialdella et al. 2006). Although no unique diagnostic feature was recorded in *Axonopus graniticola*, papillae with acute apex seems to be uncommon among species of *Axonopus* ser. *Suffulti*, being only recorded in the new species and in *Axonopus polydactylus* (Steud.) Dedecca, *Axonopus ramosus* Swallen and *Axonopus suffultus* (Mikan ex Trin.) Parodi (Cialdella et al. 2006).

#### Additional specimen examined (Paratype).

**BRAZIL: Minas Gerais:** Município Teófilo Otoni, Afloramento rochoso ao lado esquerdo da MG 418, cerca de 30 km norte de Teófilo Otoni, em inselbergue, 17°51'11"S, 41°15'44"W, 650 m alt., 16 Apr 2011, L.F.A. de Paula, M. Augsten 237 (BHCB, RB).

#### Distribution and ecology.

The new species is known only from its type locality, an *inselberg* in the municipality of Teófilo Otoni, eastern Minas Gerais, Brazil. It occurs on granitic and gneissic rock outcrops, surrounded by the Atlantic Forest matrix (Veloso et al. 1991), at elevations around 600 m.

The species is found in depressions filled with thin soil, forming dense clumps, surrounded by rocky surface. During the rainy season, the profuse growth of new leaves with conspicuous flat and wide blades gives a vivid green color to the clumps (Figure 3A), in contrast with the pale brown, almost bladeless, clumps observed during the dry season (Figure 3C). The persistent leaf sheaths covering the culms and the readily deciduous leaf blades may be adaptations to avoid desiccation during the dry season and serve as protection against high temperatures of this extremely seasonal environment. These features are described for other species among monocots families, like Velloziaceae and Cyperaceae, which are usually known as desiccation-tolerant plants (Porembski 2007, Porembski and Barthlott 2000).

The vegetation of the *inselberg* is influenced by the soil (Porembski et al. 1998, Porembski 2007), and its flora is predominantly xeromorphic. Adaptations to drought and high insolation are common for the species from the type locality of *Axonopus graniticola*. Desiccation tolerance is found in other plant groups that occur in this area, as in some ferns and allies (*Sellaginella convoluta* (Arn.) Spring, *Sellaginella sellowii* Hieron., *Cheilanthes geraniifolia* (Weath.) R.M.Tryon & A.F.Tryon) and in Angiosperms, such as Cyperaceae (*Trilepis lhotzkiana* (Nees) ex Arn.), and Velloziaceae (*Barbacenia* spp., *Vellozia* spp.). Succulence occurs in Cactaceae (*Coleocephalocerus buxbaumianus* Buining, *Pilosocereus brasiliensis* (Britton & Rose) Backeb.), Bromeliaceae (*Encholirium gracile* L.B.Sm.), Orchidaceae(*Cyrtopodium glutiniferum* Raddi, *Encyclia spiritusanctensis* L.C.Menezes), and in some Piperaceae (*Peperomia* spp.). Leaf deciduousness is also an adaptation in *Wunderlichia azulensis* Maguire & G.M.Barroso (Asteraceae) and *Tabebuia reticulata* A.H.Gentry (Bignoniaceae).

#### Conservation.

The species is known so far from a single granite-gneiss outcrop in the Teófilo Otoni region, Minas Gerais, Brazil. Due to the poor state of knowledge of the flora from that region (Martinelli 2007), more field efforts are required to clarify the distributional range of this species. In accordance with the IUCN (2001) guidelines, the species should be evaluated as Data Deficient.

Although it was not possible to assess the precise conservation status of the species, it is important to note that the vegetation of the *inselbergs* are under threat due to the ever increasing granite and gem exploration, road-building, grazing and illegal plant collection in southeastern Brazil’s *inselbergs* (Safford and Martinelli 2000). To fill the gap of information in this diverse and poorly studied area, and therefore provide guidelines for the conservation of the flora in the region, taking into account that rock outcrops support a large number of endemics (Porembski 2007), a broader study of the flora of the Teófilo Otoni *inselbergs* is urgently needed.

## Supplementary Material

XML Treatment for Axonopus graniticola

## References

[B1] Black GA (1963) Grasses of the genus *Axonopus* (a taxonomic treatment). Advancing Frontiers of Plant Science 5: 1-186

[B2] Chase A (1911) Notes on genera of Paniceae. IV. Proceedings of the Biological Society of Washington 24: 103-160

[B3] Cialdella AM Morrone O Zuloaga FO (2006) Revisión de las Especies de *Axonopus* (Poaceae, Panicoideae, Paniceae), Serie *Suffulti*. Annals of the Missouri Botanical Garden 93: 592-633.10.3417/0026-6493(2006)93[592:RDLEDA]2.0.CO;2

[B4] Clayton WD Renvoize SA (1986) Genera Graminum. Her Majesty’s Stationery Office, London.

[B5] Davidse G Judziewicz EJ Zuloaga FO (2004) Poaceae in Steyermark, J.A et. al , Flora of the Venezuelan Guyana, Vol. 8. Poaceae-Rubiaceae. Missouri Botanical Garden Press, St. Louis.

[B6] Dedecca DM (1956) As espécies brasileiras do gênero *Axonopus* (Gramineae). Bragantia 15: 251–296. 10.1590/S0006-87051956000100019

[B7] Filgueiras TS Rodrigues RS (2012)*Axonopus*. In: Forzza RC, Lista de Espécies da Flora do Brasil. Jardim Botânico do Rio de Janeiro, Rio de Janeiro, Brazil. http://floradobrasil.jbrj.gov.br/2012/FB013032/ [Accessed 24 July 2012].

[B8] IUCN (2001) IUCN Red list categories and criteria: version 3.1. IUCN Species Survival Commission. IUCN, Gland, Switzerland and Cambridge, United Kingdom.

[B9] Giraldo-Cañas D (2007) Análisis filogenético del género neotropical *Axonopus* (Poaceae: Panicoideae: Paniceae) con base en caracteres morfológicos y anatómicos. Revista Institucional Universidad Tecnológica del Chocó 29: 9-27

[B10] Giraldo-Cañas D (2008) Sistemática del género *Axonopus* (Poaceae: Panicoideae: Paniceae) y revisión de las especies de la serie *Barbigeri*. Serie Biblioteca José Jerónimo Triana 17: 1–211. Instituto de Ciencias Naturales, Universidad Nacional de Colombia, Bogotá D.C.

[B11] GPWG (Grass Phylogeny Working Group) (2001) Phylogeny and subfamilial classification of the grasses (Poaceae). Annals of the Missiouri Botanical Garden, 88: 373-457.10.2307/3298585

[B12] López A Morrone O (2012) Phylogenetic studies in *Axonopus* (Poaceae, Panicoideae, Paniceae) and related genera: morphology and molecular (nuclear and plastid) combined analyses. Systematic Botany 37: 671-676.10.1600/036364412X648625

[B13] Martinelli G (2007) Mountain Biodiversity. Revista Brasileira de Botânica 30: 587-597.10.1590/S0100-84042007000400005

[B14] Mendonça RC Felfili JM Walter BMT Silva-Júnior MC Rezende AV Filgueiras TS Nogueira PE (1998) Flora Vascular do Cerrado. In: Sano SM, Almeida SP (Eds) Cerrado: Ambiente e Flora, 289–556. EMBRAPA-CPAC, Planaltina, Brazil.

[B15] Morrone O Aagesen L Scataglini MA Diego LS Denham SS Chemisquy MA Sede SM Liliana MG Kellogg EA Zuloaga FO (2012) Phylogeny of the Paniceae (Poaceae: Panicoideae): integrating plastid DNA sequences and morphology into a new classification. Cladistics 28: 333-356.10.1111/j.1096-0031.2011.00384.x 10.1111/j.1096-0031.2011.00384.x 34836451

[B16] Porembski S (2007) Tropical inselbergs: habitat types, adaptative strategies and diverversity patterns. Revista Brasileira de Botânica 30: 579-586.10.1590/S0100-84042007000400004

[B17] Porembski S Barthlott W (2000) Granitic and gneissic outcrops (inselbergs) as centers of diversity for desiccation-tolerant vascular plants. Plant Ecology 151: 19-28.10.1023/A:1026565817218

[B18] Porembski S Martinelli G Ohlemuller R Barthlott W (1998) Diversity and ecology of saxicolous vegetation mats on inselbergs in Brazilian Atlantic Forest. Diversity and Distributions 4: 101-119.10.1046/j.1365-2699.1998.00013.x

[B19] Safford HD Martinelli G (2000) Southeast Brazil. In: Barthlott W, Porembski S (Eds) Inselbergs: biotic diversity of isolated rock outcrops in the tropics, 339–389. Ecological Studies. Springer-Verlag, Berlin.

[B20] Veloso HP Rangel Filho ALR Lima Veloso HP Rangel Filho ALR Lima JCA (1991) Classificação da vegetação brasileira, adaptada a um sistema universal. IBGE, Rio de Janeiro.

[B21] Viana P Filgueiras TS (2008) Inventário e distribuição geográfica das gramíneas (Poaceae) na Cadeia do Espinhaço, Brasil. Megadiversidade 4: 71-88

